# Microvascular changes associated with epilepsy: A narrative review

**DOI:** 10.1177/0271678X211010388

**Published:** 2021-04-17

**Authors:** Rick HGJ van Lanen, Stan Melchers, Govert Hoogland, Olaf EMG Schijns, Marc AMJ van Zandvoort, Roel HL Haeren, Kim Rijkers

**Affiliations:** 1 Department of Neurosurgery, Maastricht University Medical Center, Maastricht, the Netherlands; 2 School for Mental Health and Neuroscience, Maastricht University, Maastricht, the Netherlands; 3 Faculty of Health Medicine and Life Sciences, Maastricht University, Maastricht, the Netherlands; 4 Academic Center for Epileptology, Maastricht University Medical Center, Maastricht, the Netherlands; 5 Department of Molecular Cell Biology, School for Mental Health and Neuroscience, Maastricht University Medical Center, Maastricht, the Netherlands; 6 School for Cardiovascular Diseases, Maastricht University Medical Center, Maastricht, the Netherlands; 7 Department of Neurosurgery, Helsinki University Central Hospital, Helsinki, Finland

**Keywords:** Angiogenesis, epilepsy, microvascular density, temporal lobe epilepsy, vascular endothelial growth factor

## Abstract

The blood-brain barrier (BBB) is dysfunctional in temporal lobe epilepsy (TLE). In this regard, microvascular changes are likely present. The aim of this review is to provide an overview of the current knowledge on microvascular changes in epilepsy, and includes clinical and preclinical evidence of seizure induced angiogenesis, barriergenesis and microcirculatory alterations. Anatomical studies show increased microvascular density in the hippocampus, amygdala, and neocortex accompanied by BBB leakage in various rodent epilepsy models. In human TLE, a decrease in afferent vessels, morphologically abnormal vessels, and an increase in endothelial basement membranes have been observed. Both clinical and experimental evidence suggests that basement membrane changes, such as string vessels and protrusions, indicate and visualize a misbalance between endothelial cell proliferation and barriergenesis. Vascular endothelial growth factor (VEGF) appears to play a crucial role. Following an altered vascular anatomy, its physiological functioning is affected as expressed by neurovascular decoupling that subsequently leads to hypoperfusion, disrupted parenchymal homeostasis and potentially to seizures”. Thus, epilepsy might be a condition characterized by disturbed cerebral microvasculature in which VEGF plays a pivotal role. Additional physiological data from patients is however required to validate findings from models and histological studies on patient biopsies.

## Introduction

Epilepsy is characterized by sudden paroxysmal episodes of neuronal electric discharges, frequently provoking different types of clinically apparent convulsions, among a variety of symptoms.^1^ Epilepsy is one of the oldest known and fourth most prevalent neurological disorder, affecting up to 65 million people worldwide.^2,3^ Among neurological disorders it accounts for the highest disability-adjusted life years.^4^

To a great extent, this is due to the high rate of drug-resistant epilepsy. Drug-resistant epilepsy is defined as inadequate seizure control despite adequate trials of at least two antiepileptic drug (AED) regimes consisting of either a single drug of a combination of two or more drugs.^1,2^ In some epilepsy syndromes, like temporal lobe epilepsy (TLE), drug-resistance rates up to 38% are reported and have not decreased in recent years.^5,6^ The societal burden of chronic drug-resistant epilepsy is enormous and encompasses around 80% of total epilepsy-related costs.^4^

Despite a growing knowledge on epilepsy, current pharmacological development of 3rd and 4th generation AEDs, and accessory billions in investments over the last 15–20 years, have not led to a significant increase of seizure free patients. Hence, there is enormous need to find new therapeutic targets. Further understanding of the pathophysiology of epilepsy is therefore needed. In this regard, recent reports have emphasized the bidirectional relation between cerebrovascular diseases and epilepsy, in particular the similarities in the pathophysiology related to microvascular dysfunction.^7–11^ Unsurprisingly, Brigo et al. recently suggested that epilepsy might be the first symptom of underlying occult cerebrovascular pathology.^7^

In an attempt to elucidate the role of the cerebral microvasculature in the pathophysiology of epilepsy, we have reviewed literature on this topic. It must be noted that we will discuss the role of blood-brain barrier dysfunction (BBB) in this review to a confined extent, as many extensive reviews have been published on this topic recently.

## Angiogenesis and epilepsy

### History of studying the role of dysfunctional angiogenesis in epilepsy

In 1925, Spielmeyer, a German neuropathologist, was the first to describe microvascular abnormalities in the temporal lobe of an epilepsy patient.^9,12^ A few years later in 1934, Gibbs published on seizure-induced increased cerebral blood flow.^13^ It was only until around the last 20 years that BBB dysfunction has extensively been assessed and described by hundreds of studies.^14,15^ However, abnormalities in angiogenesis, vascular structure, and vascular function have rarely been a major topic in epilepsy research. It was not until 2007 that Rigau et al. published on microvessels and angiogenesis in epilepsy.^16^ Since then, a limited number of studies on vascular abnormalities associated with epilepsy have been published. Based on these studies, we will first describe angiogenesis and the role of vascular endothelial growth factor (VEGF) under physiological conditions, followed by epilepsy-associated abnormal angiogenesis. Thereafter, structural microvascular and functional microcirculatory abnormalities in epilepsy are discussed.

### Angiogenesis and barriergenesis: role of vascular endothelial growth factor

**Figure 1.**
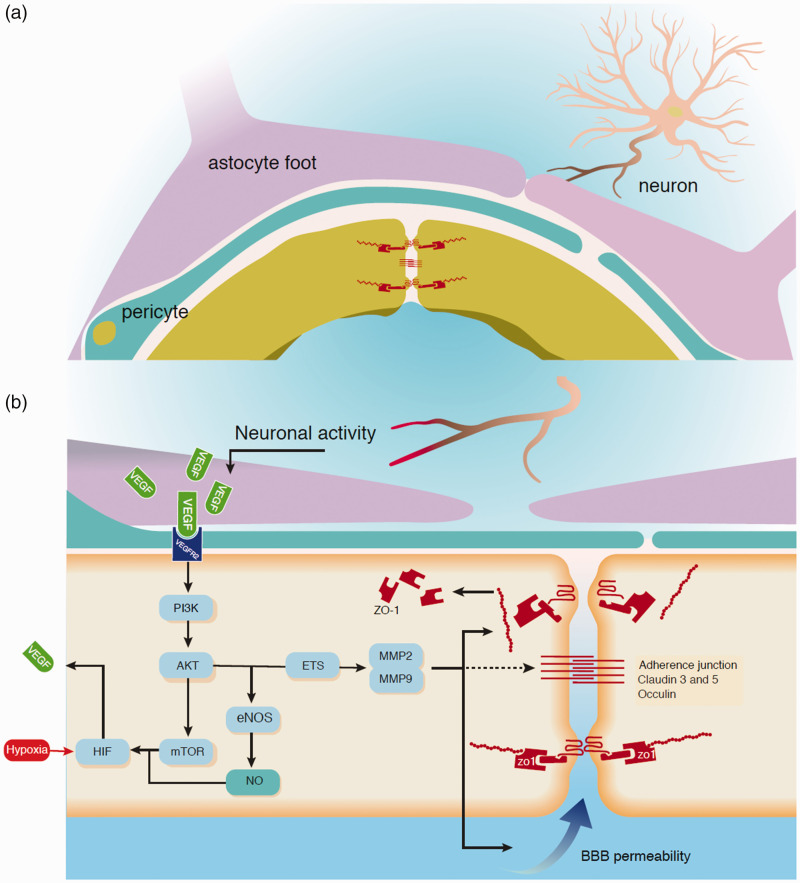
Schematic representation of processes involved in how VEGF is released and results in BBB leakage. (a) Resting situation in which VEGF is stored in large vesicles next to neurons and astrocytes. The endothelial cell barrier function is represented by adherens junctions and tight junctions formed by claudin, occludin, and ZO-1. (b) Neuronal epileptiform activity results in releasing of stored VEGF which binds to the VEGFR-2 on the endothelial cell. This activates a cascade in which PI3K and AKT are phosphorylated and activated. AKT activation results in multiple reactions. Among these are NO production, facilitated by eNOS, and mTOR activation. NO and mTOR activate hypoxia inducible factor (HIF) 1 which may be activated by hypoxia as well. HIF is a known VEGF transcription factor, therefore a positive feedback cycle of VEGF production is established. VEGFR2 activation by VEGF is known to induce matrix metalloproteinase 2 (MMP2) and MMP9, this might possibly be due to PI3K/AKT ETS-1 induction. MMP2 and MMP9 remove tight junction proteins from the cell membrane, and result in an increase in BBB permeability. The action of MMP on adherens junctions is still unknown.

In angiogenesis new vasculature is formed, under influence of angiogenetic factors, such as VEGF. This factor is represented by a group of six different dimeric glycoproteins namely VEGF-A to -F and placenta growth factor. There are three main VEGF receptors, receptor tyrosine kinases VEGFR-1 to -3. These ligands are able to bind to different receptor subtypes. VEGFR-2 has been studied mostly with its ligand VEGF-A. For a detailed review of the different VEGF isoforms, their receptors and signaling pathways, see Koch and Claesson-Welsh.^17^ VEGF is synthesized by many cells, including endothelial cells, fibroblasts, smooth muscle cells, leukocytes, neurons, and astrocytes. In the brain, VEGF-containing vesicles are observed along astrocytes and neurons.^18^ Here, VEGF is involved in neurovascular coupling and has a neuroprotective function due to the activation of certain signaling pathways.^19^ The trigger for VEGF upregulation in general is hypoxia, since hypoxia inducible factor promotes VEGF transcription,^20^ as is shown in Figure 1. Very recently, a study has been published profiling the genes responsible for this VEGF signaling in the brain of drug-resistant human TLE patients.^21^ Thirty-nine upregulated genes using the PI3K pathway as depicted in Figure 1 have been identified, confirming the relevant mediator role of VEGF signaling, thus opening up new pharmacological targets to be assessed in treatment of drug-resistant TLE.

As a master regulator of central nervous system blood vessel formation, VEGF and VEGFR-2 have received attention with regard to vascular changes in epilepsy.^22,23^ Once released, paracrine signaling involves binding of VEGF to VEGFR-2 on endothelial cells and neurons, stimulating angiogenesis. Angiogenesis includes MEK-MAPK pathway induced endothelial cell proliferation, migration, and sprouting followed by maturation.^16,24–26^ During sprouting the endothelial cells express platelet-derived growth factor β (PDGF-β) which attracts platelet-derived growth factor β-receptor (PDGFR-β), expressing abundant mural cells like pericytes and smooth muscle cells. PDGF is further discussed in a separate chapter. Interestingly pericytes form a prominent source of VEGF and therefore may induce angiogenesis themselves.^27^ Mural cell recruitment is part of the maturation process which also involves formation of tight-junctions and adherens-junctions, and generation of the extracellular matrix.^25^ Maturation involves formation of tight-junctions and adherens-junctions, recruiting mural cells (pericytes and smooth muscle cells), and generating extracellular matrix.^25^ This process results in a vessel that properly reacts to vasoactive signals and has the desired permeability.^25^ Barriergenesis, the process of vessel maturation in the brain, is even more complex, as its goal is to establish a solid BBB.^26,28^ During barriergenesis, a different subset of tight- and adherens-junctions are developed, endothelial cells are connected to the basement membrane, and mural cells cover endothelial cells.^26–28^

Barriergenesis and endothelial cell proliferation cannot be viewed as two separate mechanisms, they both occur at the same time and are initiated by their own set of physiological mechanisms.^29^ Therefore, balance between both sets of physiological mechanisms seems to be crucial for developing a healthy cerebrovascular network. VEGF’s neuroprotective role in ischemic stroke is acknowledged,^19,30–32^ but its potential pathological role characterized by vessel leakage cannot be ignored when barriergenesis lags behind endothelial cell proliferation, and leaky immature vessels are formed.^32–34^

### Angiogenesis and barriergenesis in epilepsy

The relation between angiogenesis and barriergenesis has been explored mainly in preclinical research using various rodent models mimicking TLE. As understanding these models is important for the interpretation of the results, we have summarized key features of relevant rodent models of TLE-mimicking epilepsy and techniques to study the cerebrovascular microcirculation in the online Appendix. Changes in the cerebral microvasculature, resulting from increased VEGF-induced cerebrovascular angiogenesis have been reported in epilepsy.^11,16,35,36^

### Induced VEGF release

Though is now is commonly recognized that VEGF is upregulated in epilepsy,^12,16,18,37^ it is still surrounded by many questions. What triggers this upregulation, what cells are responsible, and how is this release mediated, remains to be elucidated.

Hypoxia is the main trigger for VEGF upregulation in general, but it is unclear if this mechanism also applies to epilepsy. On the one hand, hypoxia-inducible factor 1a (HIF-1a) has been shown to be co-expressed with VEGF in human temporal cortex and hippocampal tissue,^38,39^ as well as in the coriaria lactone rat model,^39^ and in the lithium-pilocarpine-induced status epilepticus rat model.^40^

On the other hand, tissue oxygen levels in epilepsy patients appear normal during a seizure.^18,37,41^ Hypoxia-induced VEGF upregulation may otherwise occur in long lasting status epilepticus (SE), but most TLE patients never experience such a life-threatening SE. Furthermore, rodent electroconvulsive seizure (ECS) model studies, a model for electroconvulsive treatment, have shown that VEGF release is not affected by hippocampal oxygen levels.^42^ Apart from being a possible VEGF releaser, HIF-1a is also known to mediate apoptosis,^40^ which may explain its detection in the above-mentioned studies. Compared with excitotoxicity, the relative contribution of hypoxia to the development of HS is considered larger in animal models than in human epilepsy.^43^ Thus, the mechanism of hypoxia-induced HIF-1a expression that consequently induces VEGF upregulation seems to play a role in human epilepsy. In this regard, neuronal activity on itself may form an alternative stimulus for VEGF upregulation.^12^ This is supported by the finding that administration of tetrodotoxin (TTX), a neuronal activity suppressor, in kainate-treated hippocampal cultures prevented upregulation of VEGF,^18^ suggesting that some form of neuronal hyperactivity was necessary. However, a critical contribution of local hypoxia by prolonged postictal hypoperfusion to VEGF upregulation at the level of microcirculation cannot be excluded.^44,45^ Furthermore, experimental SE is associated with increased neuronal and astrocytic VEGF expression immediately after SE,^18^ and one week later.^37^ Similar findings on VEGF^16,46^ and VEGFR-2^18^ were noted in resected hippocampi from TLE patients as well as in the rodent ECS model.^47^ Thus far, little is known on the origin of this increased VEGF, but in the chronic phase of experimental epilepsy in rodents, reactive astrocytes have been found to be the main source.^16^ Moreover, in vitro reactive astrocytes can directly promote endothelial cell proliferation using VEGF.^48^

### Angiogenesis increases vessel permeability

VEGF also affects vessel permeability by temporarily increasing leakiness of functioning mature vessels following VEGF release.^25,49^ This phenomenon has also been observed in epilepsy. VEGF release was associated with the loss of the tight junction protein ZO-1 (Figure 1),^18^ while neutralizing VEGF decreased vessel leakiness and angiogenesis.^11^ The loss of ZO-1 may be directly involved in increased vessel permeability and is the result of two VEGF induced pathways. One being by the VEGFR-2/Src pathway in which phosphorylation of the tyrosine kinase Src directly downregulates ZO-1.^18^ The other one being by activation of endothelial cell transcriptional regulator (ETS-1). ETS-1 stimulates the synthesis of matrix metalloprotease 2 (MMP2) and matrix metalloprotease 9 (MMP9), which leads to the degradation of ZO-1,^50–52^ resulting in increased BBB permeability.^53,54^

**Figure 2.**
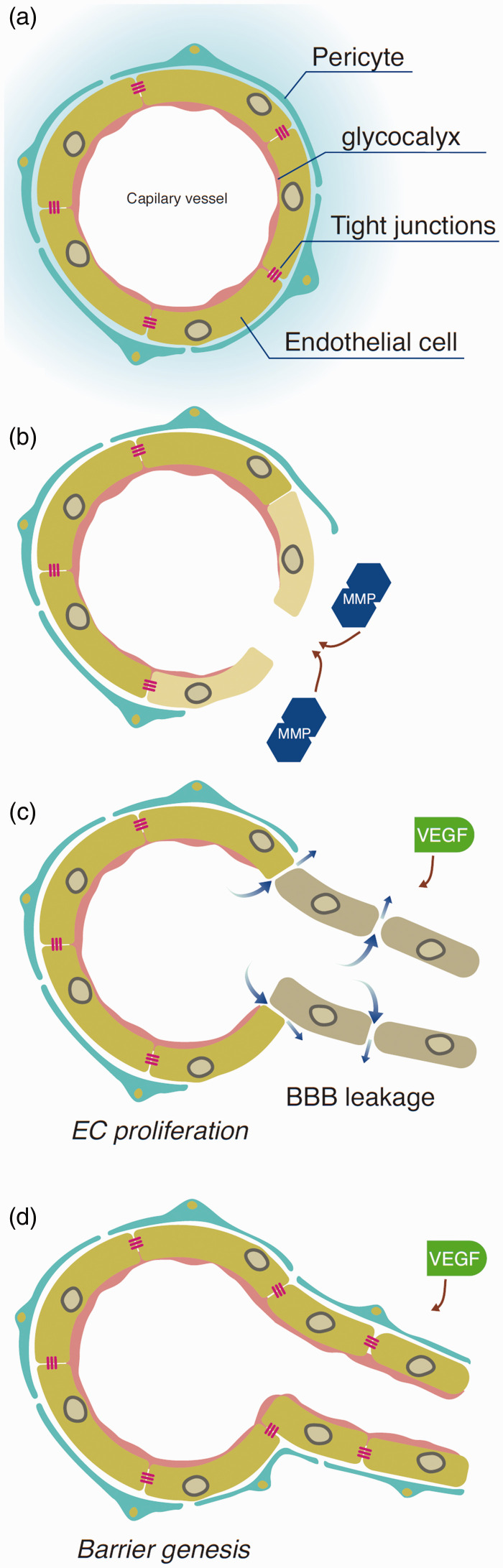
Schematic illustration of the possible misbalance between endothelial cell proliferation and barriergenesis. (a) Resting state in which the endothelial cells are covered with basement membrane (BM), pericytes imbedded in the BM and linked to each other with adherens- and tight-junctions. (b) MMP’s result in BM and junction breakdown. (c) VEGF, along with other factors, results in endothelial cell (EC) proliferation. (d) Barriergenesis restore the BM and pericyte coverage and junctions are formed. Excessive VEGF might result in a misbalance between (c) and (d), therefore newly formed vessels might have increased BBB permeability.

Increased angiogenesis can also be harmful when maturation and barrier development lag behind. Macular degeneration, an ocular condition, is an excellent example of this. In the early stage, hypoxia of the retina occurs due to reduced perfusion.^55^ This leads to excessive VEGF activity, resulting in enhanced angiogenesis and many newly formed, but leaky vessels: a leaky blood-ocular-barrier. The subsequent extravasation is successfully reduced by local anti-VEGF injections.^55,56^ Since the blood-ocular-barrier is quite comparable to the BBB, these findings may be extrapolated to the situation in epilepsy patients. As stated, neuronal activity on itself may form an alternative stimulus for VEGF upregulation, however, relative hypoperfusion as a result of more pronounced vasoconstriction in pial arteries from epilepsy patients has been described in literature.^44,45^ Furthermore, blood-oxygen-level-dependent magnetic resonance imaging abnormalities have been appreciated, indicative of perifocal hypoperfusion.^57^ The process of hypoperfusion will be explained in depth when discussing microcirculation. These processes set in motion mechanisms whereby leaky vessels develop in the brain (Figure 2) due to a misbalance between barriergenesis and endothelial cell proliferation. Indeed, in both TLE patients and animal epilepsy models, the formation of basement membrane protrusions or string vessels are observed, supporting this hypothesis.^28,29^ The phenomena of basement membrane protrusions and string vessels are further discussed in more detail in the vascular morphology section.

### Blood brain barrier dysfunction in epilepsy

The major structural barrier of the BBB resides in endothelial cells, containing intercellular tight and adherence junctions, lack intercellular fenestrations, and are characterized by low pinocytotic activity. Thus, the BBB largely function as a diffusion barrier, limiting paracellular movement through cerebral endothelial cells. Tight junctions consist of transmembrane proteins, including occludin, claudins, and junction adhesion molecules. Adherence junctions are composed of protein complexes such as cadherins and catenin.^26^ It has been shown consistently that in epilepsy, BBB permeability is increased.^58^ This BBB leakage has been related to a number of structural abnormalities.

Degradation of the lamina basalis and tight junctions have been observed.^59^ Surgically resected brain tissue of epilepsy patients has been reported to contain both increased and decreased levels of the main structural components of tight junctions such as claudins (especially claudin-5) and occludin.^60^ Adherence junctions and their associated proteins have furthermore been implied in the onset and progression of epilepsy in animal studies.^61^ In addition, matrix metalloproteinases (MMPs) are an important player in extracellular matrix remodeling, and contribute to a large variety of brain disorders by their involvement in inflammatory responses and BBB disruption by mediating the loss of basal lamina proteins.^22,54^ A recent study identified hub genes underlying epileptogenesis in TLE.^62^ These genes mainly participate in epileptic pathophysiology, including inflammation, BBB damage, cell adhesion, microglia/macrophage activation, and activation of complements. This study further found that some hub genes in human TLE positively correlated with seizure frequency and HS.^62^

BBB leakage in epilepsy is triggered by seizures and activates a pathway that involves glutamate signaling through cytosolic phospholipase A_2_, which increases MMP levels and decreases tight junction protein expression levels and breakdown of the basement membrane.^54^ Furthermore, BBB leakage leads to the presence of large serum proteins such as albumin in the parenchyma.^16^ For example, exposure of rodent cortex to bile salts, results in detection of extravascular albumin, indicating BBB opening.^63^ It also led to the generation of an epileptic focus.^63^ Albumin in the parenchyma can bind the transforming growth factor β 2 receptor on astrocytes.^22^ Upon binding, DNA transcription is altered, leading to astrocytic transformation and ultimately dysfunction.^58^ These transformed or activated astrocytes are prominent in the epileptic brain, and are known to reduce seizure thresholds.^64^

Interestingly, this BBB leakage has been visualized by multimodal imaging rats in the early phase of SE-induced epileptogenesis.^65^ Positron emission tomography, single photon emission computed tomography and magnetic resonance imaging all enabled sensitive detection of BBB disturbances during epileptogenesis, as validated by post-mortem detection of extravascular albumin.^65^ In vivo visualization of the BBB integrity may advance the understanding and potential treatment of BBB permeability in human epilepsy.

Previously, we discussed the possible relationship between seizure frequency, vascular density^16^ and increased levels of VEGF and its receptor,^60^ contributing to typical vascular injuries seen in epileptic foci, such as edema, inflammation and increased vessel permeability, contributing to BBB dysfunction. As angiogenesis affects the local vascular network, it subsequently triggers release of neuroinflammatory factors, leading to neuronal scarring, promoting atrophy and seizure progression.^66^ In line with this observation, recent clinical data have shown a relationship between MVD and malformation of cortical development, focal cortical dysplasia and tuberous sclerosis complex.^67^ These cortical developmental abnormalities are among the most common causes for epilepsy.^68^

### PDGF-β and epileptogenesis

Pericytes cover approximately 60-80% of the cerebral microvasculature, are connected to endothelial cells by adherens junctions, and thereby are an indispensable part of the BBB. Due to their morphology and location, pericytes can simultaneously signal to multiple endothelial cells, sense hemodynamic forces within the vessel, and play a role in maintaining central nervous system homeostasis. It is assumed that they could be key players in barriergenesis. Concerning barriergenesis, under physiological conditions there is an equilibrium in turnover of pericytes in the vessel wall and basement membrane.^69^ Besides pericytes, PDGFR-β is also expressed by stromal cells like fibroblast, oligodendrocyte precursor cells and endothelial cells. In case of SE or seizure-like events, the turnover of pericytes is disrupted, reflected by a decrease in pericyte cell number and a disarranged appearance. Interestingly, in these cases PDGFR-β expression is increased.^69,70^ Also, in the first week after SE, PDGFR-β expression mainly accumulates in areas undergoing extensive neuronal damage like the hippocampal CA3 region.^71^ This suggests that PDGF-β either causes neuronal damage itself, or that it plays a role in tissue repair to counterbalance the initial loss of pericytes and thereby restores the barrier function.

A second role for PDGF-β in epilepsy is related to pericyte-glia scar formation. The inhibition of PDGFR-β in vitro reduces fibrosis and may thereby attenuate pericyte-glia scar formation.^10^ Indeed, pericytes embedded in the vascular wall are able to detach from the basement membrane and then adopt a microglial phenotype.^71^ However, microglial-like cells expressing PDGFR-β remain to be demonstrated in clinical and experimental epilepsy.^71,72^ Moreover, treatment with a PDGFR-β agonist post-SE in a kainate rodent model of epilepsy mainly affected barrier restoration, it resulted in a reduced pericyte loss and attenuated BBB-leakage.^69^ Increased PDGFR-β reactivity has been found in hippocampi resected from TLE patients with HS, showing that PDGF-β indeed plays a role in TLE.^70,72^ Collectively, these studies indicate that seizures result in increased PDGFR-β expression. PDGFR-β could play an anti-epileptogenic role consisting of BBB-restoration or restoration of neuronal damage, or may be epileptogenic by contributing to pericyte-glia scar formation and/or neuronal damage. The predominant effect is and whether this effect is for example concentration and/or time dependent has yet to be scrutinized.

### VEGF and epileptogenesis

Besides a clear increase in VEGF expression in chronic epilepsy, a potential role for VEGF during epileptogenesis is suspected as well, and anti-VEGF therapy has been implicated.^32^ For example, the VEGFR-2 inhibitor sunitinib completely prevents angiogenesis, HS, and seizures induced by pilocarpine.^73^ Whether this suggests a potential novel antiepileptic treatment is debated, because sunitinib comes with side-effects. It inhibits other tyrosine kinase-dependent pathways, like the PDGF-β/PDGFR-β signalling and nitric oxide produced inhibition.^74^ Therefore, the positive results may not completely be due to VEGF inhibition but also to additional inhibitory effects of sunitinib.

Other VEGF or VEGF-signaling inhibitors have similar effects. Rapamycin diminishes both seizures and HS by inhibition of mTOR, a kinase in the positive feedback loop of VEGF (Figure 1).^75^ This inhibition leads to reduced BBB leakage and reduced activation of microglia and macrophages.^75^ In kainate-treated hippocampal slices, anti-VEGF administration (by rat VEGF antibody AF564) resulted in less vascular abnormalities, most profoundly seen as a lack of branching in CA1 and CA3.^18^ The effect of anti-VEGF on epileptiform activity in this model was not reported.

The effects of the VEGF signaling pathways are not solely pro-epileptogenic. Besides vascular effects, VEGF can also trigger proliferation, differentiation and migration of neuronal progenitor cells and astrocytes by acting on their VEGFR-2. On neurons this is shown by an increased outgrowth of neurites and survival.^76^ In epilepsy the latter is achieved by signaling through the PI3K/Akt pathway that protects against excitotoxicity.^59,77^ Like its effect on neuronal progenitor cells, the VEGFR-2 controls migration of oligodendrocyte progenitor cells. To illustrate the diverse effects of the VEGF-family; VEGF-C stimulates proliferation of oligodendrocyte progenitor cells through VEGFR-3.^76^ Furthermore, increased VEGF-induced vascularization and initial neurovascular coupling by the VEGF-eNOS pathway provides sufficient perfusion in high energy demand areas.^59^ Other studies have found these positive effects of VEGF as well: in a study on pilocarpine-treated animals, VEGF administration appeared to completely prevent seizure induced neuronal loss in the CA1 region. Interestingly, no vascular changes were observed, while seizures continued. However, these findings were observed during the acute-phase.^78^ In another study from the same research group, the neuroprotective effects of VEGF administration were lost when testing the animals 4 weeks post-SE.^79^ This suggests that the negative effects of VEGF may outlast the positive in the long run.

In summary, epileptic seizures are linked to a misbalance in VEGF homeostasis. Neuronal activity and reactive astrocytes may form an alternative pathway for VEGF release and induced angiogenesis, possibly in the absence of hypoxia. This VEGF release induces a misbalance between barriergenesis and endothelial cell proliferation, leading to the formation of functionally mature, but leaky vessels. This would result in disturbed angiogenesis, abnormal microvascular morphology and microvascular density (MVD), and BBB dysfunction. Indeed, such abnormalities have been described in literature; BBB dysfunction has been extensively studied, and numerous reviews have already been published on this topic.^14,15^ Therefore, we present an overview of findings on microvascular density and morphological changes in epilepsy.

## Microvascular abnormalities in epilepsy

Most studies on microvascular abnormalities in epilepsy have been carried out using animal models. The models used in these studies vary considerably. To better understand and value results from these studies, we will first very briefly discuss these different epilepsy models. A more extensive discussion on these models can be found in the online Appendix and Supplemental Table 1 and 2. Subsequently, the vascular abnormalities found in these studies are described. Techniques and methods to evaluate microvascular structures and their utility are presented in the online Appendix.

### Epilepsy models

Several models have been used in literature to mimic and study epilepsy or seizures, including post post-SE models, kindling,^46,80^ rodent ECS models,^42,80,81^ and organotypic hippocampal cultures (OHCs).^18^

In post-SE models, like pilocarpine^11,16,35–37,73,78,82^ and kainate^48,75^ models, three subsequent phases of epileptogenesis can be distinguished: acute, latent, and chronic. The acute phase, consisting of an induced SE and physical recovery, is followed by a latent phase where no behavioral seizures are observed, though non-convulsive seizure events can occur, and a chronic phase where spontaneous recurrent seizures occur.^83–85^ Long-term continuous video-EEG recordings from neocortex and hippocampus show that kainate-treated rats usually begin to have non-convulsive seizures approximately 1 week after kainate treatment.^86^

In the kindling model, the brain is stimulated by either a chemical substance or electrical stimulus which initially provokes a subtle focal seizure. By repetitive stimulation seizure threshold decreases leading to gradual induction of seizures of increasing severity, and ultimately to spontaneous seizures.^86^

Another model to study seizures is ECS, in which electroconvulsive therapy is modelled by provoking seizures by applying an electric current through the brain. This can be the whole brain, or specific parts, such as hippocampal electrical stimulation.

Finally, OHCs are hippocampal neurons that can be maintained within their neuronal network in in-vitro for a limited amount of time. In this model, a latent phase without electrical activity is followed by spontaneous electrical activity with epileptiform characteristics.^87^

## Microvascular density alterations in epilepsy models

Since most studies reporting on microvascular abnormalities discuss microvascular density (MVD) as an important structural alteration in epilepsy, we will first discuss MVD. It must be noted that vascular density has not been assessed and quantified uniformly, making comparisons and drawing conclusion more challenging.

### Limbic MVD changes in post-SE models

Several studies on MVD using kainate or pilocarpine models have been published. The most important effects these studies observed during three different phases (acute, latent, and chronic) are summarized in Table 1. Both an increased and decreased MVD, varying from 80–140% of control have been reported.

**Table 1.** Changes in microvascular density of chemically induced epilepsy in rodent models on microvascular density during the latent and chronic phase.

Study	A	Seizure paradigm	Stain method	Quantification method	Timing (days)	Study population	Number of controls	Effects on microvascular density in different regions						
								Cortical	Amygdala	CA1	CA2	CA3	DG	Mean hippocampus
Rigau et al. 2007	R	Pilocarpine i.p.	DAB and haematoxylin	Point-counting	4–14	11	7	–	–	–	–	–	–	↑
					21–28*	7	7	–	–	–	–	–	–	↑
Romariz et al. 2013	M	Pilocarpine i.p.	Anti-laminin	Fluorescent microscope and analysis	7	7	10 total β	–	–	–	–	–	–	NS
					15*	7	10 total β	–	–	–	–	–	–	NS
Zhai et al. 2015	M	Kainate i.p.	Anti-CD31	CD-31 + percentage area	3	?	?	–	–	–	–	–	–	↑
					5	?	?	–	–	–	–	–	–	↑
					7	?	?	–	–	–	–	–	–	NS
Benini et al. 2016	R	Pilocarpine i.p.	Isolectin B4 and DAPI	Endothelial cell count/mm^2^	22*	6	3	–	–	↑	↑	↑	–	–
Hayward et al. 2010	R	Pilocarpine i.p.	RECA-1	Point-counting	14*	6	5	NS	↑	–	–	–	–	–
Marcon et al. 2008	R	Pilocarpine i.p.	Anti laminin	Point counting	7	5	5	↑	↑	–	–	–	–	↑
					90*	5	5	↑	↑	–	–	–	–	–
			FICT-albumin	Point counting	7	5	5	↑	↑	–	–	–	–	↑
					90*	5	5	↑	↑	–	–	–	–	–
Van Vliet et al. 2016	R	Kainate i.p.	RECA-1	Microscopy scoring scale	49*	6	4	↑	↑	–	–	–	–	–
Feng et al. 2016	M	Pilocarpine i.p.	Anti CD31	2D plane imaging	7	6	6	–	–	NS	NS	NS	–	–
					14	6	6	–	–	NS	NS	NS	–	–
					28*	6	6	–	–	↑	NS	↑	–	–
					56*	6	6	–	–	NS	NS	NS	–	–
Ndode-Ekane et al. 2010	R	Pilocarpine i.p.	RECA-1	Virtual sphere method	2	6	2–3	–	–	↓	–	NS	–	↓
					4	5	2–3	–	–	NS	–	NS	–	NS
					14*	14	2–3	–	–	NS	–	↑	–	↑

* = timing of measurements executed during the chronic phase. Timing expressed in days post SE.

A = animal, M = mice, R = rats, i.p. = intraperitoneal, ↑ = increased, ↓ = decreased, NS = not significant, (–) = not determined, DG = Dentate gyrus, DAB = 3’,3’ diaminobenzidine. β = not stated at what time point the control rats were offered.

SE models consist of a long-lasting SE, which on itself can result in local hypoxia and therefore induce angiogenesis. However, during the acute phase, no alterations in MVD have been reported (see Supplemental Table 3),^16,36,48^ which may suggest the hypoxia caused by SE does not immediately contribute to MVD changes. In the subsequent latent phase, inconsistent changes in MVD were noted (Table 1). Ndode-Ekane et al. reported on MVD decrease in the early latent phase (2 days after SE) in CA1 which returned to baseline in the latent phase (4 days).^11^ The relatively short interval of 2 days raises the question whether this MVD decrease accompanied SE itself, or epileptogenesis. Two other studies did not find any changes in MVD in limbic regions at 4 and 7 days.^35,36^ Conversely, three studies report a clear significant MVD increase (p-values two <0.01 and one <0.05) in the hippocampus at 3–14 days after SE.^16,37,48^ Only one of these three papers investigated the amygdala, and found an increase there as well.^37^

In the chronic phase, an increase in MVD has been reported on frequently in both the amygdala and hippocampus,^11,35,37,73,75,82,88^ whereas Romariz et al. did not observe any significant changes.^36^ This may be due to the fact that they used anti-laminin as a vascular marker. This marker does not stain the endothelial cells itself. Instead, it stains the basal lamina which, explained by the endothelial cell proliferation - barriergenesis mismatch, may not follow the endothelial cell proliferation one-to-one. Interestingly, Feng et al. showed an initial MVD increase at 28 days post-SE, recovering to control levels at 56 days.^35^ They proposed that angiogenesis is most prominent during the first days after SE and slowly regenerates to control levels. This is supported by the fact that RECA-1/BrdU, a marker for endothelial cell proliferation, is considerably increased during the latent phase of pilocarpine-induced seizures in rats.^11^

### Limbic MVD changes in chemical kindling models

Chemical kindling using intra-hippocampal sulfoximine (MSO)^80^ or intraperitoneal pentylenetetrazol (PTZ)^46^ has been conducted in rodents. Since these models do not provoke a massive SE, the hypoxic conditions are limited. No changes in MVD were observed in these rodents at 28 and 22 days, respectively (see Supplemental Table 4). This might be related to the kindling technique, as it is a long-term process where generalized seizures are only observed after a certain number of injections.^46^ In this specific model, structural changes may hypothetically only appear after an even longer period of time: Hence, assessment of MVD 22–28 days after start of kindling may have been too early. Since kindling models seem more representative of the clinical situation, long-term research on MVD in kindling based epilepsy models is desired.

### Limbic MVD changes in ECS models

ECS involves electrical stimulation of the brain to mimic electroconvulsive therapy. It does not involve the introduction of neurotoxins. Like in epilepsy, ECS is associated with enhanced BBB permeability, astrocyte activation, and neurogenesis.^89^ However, unlike epilepsy, no apoptosis, HS, or disruption of tight junctions are found following ECS.^89,90^ Therefore, results from ECS studies can be interesting by providing new insights into possible brain (patho) physiology, but cannot directly be extrapolated to understanding of the pathophysiology of epilepsy. To some extent ECS and electrical stimulation-induced kindling are both epilepsy models, since they are both characterized by repetitive seizures.^91^ However, the ECS and kindling model differ with respect to the inter-seizure interval, i.e. in ECS the full-fledged therapy is given on day one, while in electrical simulation-induced kindling models this is administered over several consecutive days. Thus, ECS may be somewhat comparable to induced SE, where cerebrovascular remodeling starts immediately after the first day of stimulations.

Three papers on electrically induced seizures and limbic MVD have been published, two using ECS^42,81^ and one using hippocampal electrical stimulation-induced SE^80^ (Table 2). Two ECS studies followed the same ECS protocol consisting of ECS once daily for 10 days,^42,81^ but the interval between the final ECS seizure and sacrifice was 1 day in one study^81^ and 11 in the other.^42^ Nevertheless, both studies described an increase in MVD, though in different regions of the hippocampus (CA1 vs DG). We found one other paper on ECS in rats, describing increased endothelial cell proliferation (measured by BrdU-RECA-1 co-expression) 7 days after ECS initiation. The result of increased endothelial cell proliferation, leading to vascular remodeling and increased MVD, might be observed if this process continues for a certain period of time, thus showing similarities with MVD findings in other epilepsy models. However, as MVD was not measured we did not include this study.^92^ Hippocampal MVD remained unchanged in the paper on hippocampal electrical stimulation-induced SE.^80^ Unfortunately, interval between SE and time of sacrifice was not reported in this paper.

**Table 2.** Changes in microvascular density of electroconvulsive treatment in rodents.

Study	A	Seizure paradigm	Stain Method	Quantification Method	Timing	Study population	Number of controls	Effects on microvascular density in different regions						
								Cortical	Amygdala	CA1	CA2	CA3	DG	Mean Hippocampus
Chen et al., 2018	R	O.D. 55-70mA given via clip electrodes in 0.5 s at 100 Hz for 10 days.	Thionin staining	Global spatial sampling method. Only 1 celled vessel with diameter ≤10 μm are measured	15h after last ECT treatment	6	6	–	–	↑(stratum radiatum)	–	–	–	–
Hellsten et al. 2005	R	O.D. 50 mA, 5 sec, 50 Hz bilateral ECS treatment via ear clips.	RECA-1	Global spatial sampling method	11d after last ECT treatment	6	6	–	–	–	–	–	↑	–
Lauritzen et al., 2012	R	Bilaterally electrode implants in angular bundle of performant pathway and in the dentage granule cell layer. 3 or 8 hour stimulation. Isoflurane gas admission to terminate residual seizure activity	RECA-1	Point count	Unclear* *= several days after continuous video-EEG recordings the rats were offered	5	5	–	–	NS	–	NS	NS	–

ECT = electroconvulsiontherapy. O.D. = once daily, A = animal, M= mice, R = Rats, ↑ = increased, ↓ = decreased, NS = not significant, (–) = not determined. DG = Dentate gyrus.

### OHC models

One study used kainate directly on organotypic hippocampal cultures obtained from rats (summarized in Supplemental Table 5).^18^ Hippocampal MVD was increased during the recovery period when cultures were perfused with kainate-free solution. This model suggests that angiogenesis continues even when the cells are no longer exposed to the chemical substance. However, data obtained form OHC-models should be interpreted with care since homeostasis and BBB function are compromised due to the lack of regular blood flow. Besides, cultures are maintained in an artificial growth medium which is likely to affect physiological development into histopathological features of epilepsy in the hippocampus.^86,87^

### Cortical MVD changes in epilepsy models

Three papers, using kainate and pilocarpine models, reported on neocortical MVD.^37,75,82^ Two studies showed a significant (p < 0.05) MVD increase during the latent and chronic phase in pilocarpine and during the chronic phase in kainate (Table 1).^37,75^ The third study found no difference during the chronic phase in pilocarpine.^82^

## Microvascular alterations in human epilepsy

Histological examinations of MVD in humans can either be performed using post-mortem brain tissue or by analyzing surgically resected tissue. The latter can be performed in all patients undergoing resective brain surgery. It must be noted that tissue from epilepsy patients represents the chronic state of epilepsy as end-stage disease, and not the process of epileptogenesis. Unfortunately, it is also extremely difficult to obtain human hippocampal control (non-epileptic) tissue. The vast majority of for example low grade glioma patients requiring resective brain surgery suffer from epilepsy, especially if the lesion has a mesiotemporal location. Instead, post-mortem hippocampal tissue obtained during autopsy is used as surrogate control, which comes with the disadvantage of a longer post-mortem interval compared to fresh-frozen tissue after surgery. In addition, post-mortem tissue is obtained from on average older patients compared to epilepsy patients undergoing resective epilepsy surgery. These differences must be taken into account when comparing post-mortem tissue to tissue from epilepsy patients.

### Hippocampal MVD alterations in TLE patients

Five papers studying hippocampal MVD in TLE patients are described in detail in Table 3. Two of these papers reported on a significant MVD (p < 0.01) increase,^16,72^ one reported on unchanged MVD,^93^ and two reported on a significant (p < 0.01) MVD decrease.^94,95^ The studies which reported on increased MVD described a locoregional increase restricted to one (mostly CA1) of the hippocampal subfields,^72^ or an overall increase within the hippocampus up to 224% of control.^16^ Increase of MVD was strongly correlated with seizure frequency.^16^ However, a small study on two resected post-mortem TLE hippocampi,^93^ using the same staining method as Garbelli et al,^72^ did not find this MVD change.

**Table 3.** Microvascular density findings in humans with TLE.

Study	Patients	Controls	Stain method	Quantification	Finding of microvascular density in region:							
					Subiculum	Cortical	CA1	CA2	CA3	CA4	DG	Hippocampus
Liu et al., 2012	Post mortem TLE with HS (n = 2)	Control post mortem, no neurological diseases known (n = 4)	Anti-CD34	CD-34+ percentage area	–	NS (temporal cortex)	–	–	–	–	–	NS
Garbelli et al. 2015	Surgery samples with HS (n = 4)	Peritumoral cortices, distant from lesions. No epilepsy in history. (n = 4)	Anti-CD34	Not explained in paper	–	–	↑	–	↑	↑	–	–
Mott et al., 2009	TLE patients undergoing temporal lobectomy (n = 4)	Autopsy brains free of significant pathologic changes (no epilepsy noted) (n = 3)	Anti-type-IV-collagen Anti-AP	AP+ and anti-type-IV collagen+ percentage area	–	–	NS	↓	NS	NS	NS	↓
	TLE patients undergoing temporal lobectomy n = 6	Autopsy brains free of significant pathologic changes (no epilepsy noted n = 3	Anti-type-IV-collagen Anti-AP	Global spatial sampling method	–	–	NS	NS	NS	NS	NS	NS
Rigau et al., 2007	TLE patients undergoing temporal lobectomy. Without HS. (n = 9) And with HS (n = 8) Total (n = 17)	Autopsy patients without neurological diseases. (n = 3) And patients undergoing hippocampectomy for a tumorous process. (n = 2) Total (n = 5)	Anti-Von Willebrand factor	Point-counting method	–	–	–	–	–	–	–	↑
Kastanauskaite et al., 2009	TLE patients undergoing surgery temporal lobectomy with HS (n = 19)	Autopsy patients without any neurological diseases. (n = 5) and TLE patients undergoing surgery temporal lobectomy without HS. (n = 5) Total (n = 10)	Anti- type- IV-collagen	Collagen+ percentage area	–	–	↑	–	–	–	–	–
		Autopsy patients without any neurological diseases. (n = 5)	Anti-AP	AP+ percentage area	NS	–	↓	NS	NS	NS	NS	–
		Autopsy patients without any neurological diseases. (n = 5) and TLE patients undergoing surgery temporal lobectomy without HS. (n = 5) Total (n = 10)	Toluidine blue	Point counting measuring volume	–	–	↓	–	–	–	–	–
	TLE patients undergoing surgery temporal lobectomy without HS. (n = 5)	Autopsy patients without any neurological diseases (n = 5)	Anti-AP	AP+ percentage area	NS	–	NS	NS	NS	NS	NS	–

AP = alkaline phosphatase, TLE = Temporal lobe epilepsy, HS = Hippocampus sclerosis, **↑** = increased, ↓ = decreased, NS = not significant, (–) = not determined, DG = Dentate Gyrus.

Two human studies reported on MVD decrease. In one study with four TLE patients undergoing temporal lobectomy, the overall hippocampal MVD decrease to 88% of control MVD values was mainly related to a strong decrease in the CA2 region.^95^ However, by applying a different MVD determination method, no significant change (106% compared to control MVD) was observed (see for additional information Supplemental Table 2).^95^ The other study reported on reduced MVD restricted to CA1, causing overall density to be decreased to 60% of control MVD. This decrease in CA1 was only found in the 19 TLE patients where HS was histopathologically confirmed.^94^ In other hippocampal regions, no MVD changes were reported.

These findings are in contrast with 2 studies reporting an increase in MVD,^16,72^ which may be due to difference in staining: both studies reporting on MVD decrease used alkaline phosphatase (AP) and a collagen-IV stain, while the MVD papers detecting an increase used anti-Von Willebrand or anti-CD34. AP is a vessel marker staining afferent vessels like small arterioles. Consequently post-capillary venules and small veins are not taken into account using this technique.^95^ Both anti-Von Willebrand and the anti-CD 34 are antibodies visualizing endothelial cells, thus staining all blood vessels. Collagen-IV stain visualizes the basement membrane, which is also present in all vessels. In one of the MVD decrease papers, the basement membrane appeared to be increased up to 258% of the normal value.^94^ The paradox of decreased AP staining using collagen-IV is ascribed to the presence of string vessels, which will be discussed in the paragraph on “morphological changes in epilepsy”. In conclusion, humans suffering from epilepsy show an increased, decreased or unchanged MVD. In either case, changed MVD may be restricted to either afferent or efferent vessels. Moreover, a MVD decrease seems to be associated with pathological abnormalities such as HS^94,95^ and FCD.^72^

### Cortical MVD alterations in TLE patients

In humans, one study reported on MVD changes in the neocortex of two TLE patients with HS, and were compared to four post-mortem controls. No significant difference was found (Table 3).^93^ Structural changes in the cerebral cortex, like cortical surface area reduction, are reported in TLE patients.^96^ More focus on the neocortex is desirable because the neocortex is frequently involved in TLE as well as extra-temporal lobe epilepsy. Seizure spread from limbic to temporal neocortical structures are found to be accompanied by morphological changes in the neocortex of operated epilepsy patients.

## Vascular morphology in epilepsy models

Several studies assessing MVD encountered some interesting morphological changes in both rodent (pilocarpine,^11,35,88^ kainate^10^ and ECS^42^) and human hippocampi.^16,94,95^ These findings are summarized below.

### Vascular morphological changes in animal models

In rodents, the earliest structural microvascular change reported is increased vascular branching in CA1 and CA3. This was observed as early as during the acute phase in pilocarpine^35^ and continues during the latent and chronic phase in the same model.^11^ This branching appeared to have led to a disorganized microvasculature around the pyramidal cells of CA1 and CA3 during the chronic phase.^11,35^ In healthy individuals, vessels normally emanate from the fissure (stratum radiatum) perpendicular to the pyramidal cell layer in the CA1 and CA3 areas as Y- or T-shaped.^11,35^ In epilepsy models, this characteristic perpendicular appearance in the pyramidal layer is replaced by a more or less transverse entrance into this layer.^11,35^ Besides a difference in organization, structural changes have been observed as well. Distorted, fibrous, irregularly oriented, and fragmented vessels are observed in the pilocarpine model.^35,88^ This finding was confirmed with laminin sprouts devoid of RECA-1 staining in the molecular layer of rodents who underwent ECS.^42^ This finding suggests that there is protrusion of the basal lamina without endothelial cells following. However, in the kainate model, depositions of collagen-III and –IV, both physiologically present in basal lamina, are found in CA1 and CA3. These depositions are found in the latent phase as well as in the chronic phase and are suggested to be part of a perivascular scar formation.^10^

Two papers specifically reported on vessel diameter changes in pilocarpine.^11,35^ An increase in vessel diameter in CA1 and CA3 was found in the acute phase,^11^ and the transition phase between latent towards chronic, that evened out during the chronic phase.^35^ No accompanying MVD change was observed. Interestingly, a highly and significantly increased number of thrombi was found compared to control rats at this time-point as well.^11^ The number of thrombi as well as vessel diameter slowly decreased during the latent phase. To the best of our knowledge, no other reports on (micro)thrombus formation in epilepsy have been published in literature. Therefore, formation of thrombi may be an epiphenomenon of the model, but on the other hand it could be an interesting finding that might explain the increased risk of cerebrovascular disease in epilepsy patients.

## Vascular morphological changes in human epilepsy

Similar structural and organizational microvascular changes as described in rodents, are observed in human hippocampi: TLE patients’ hippocampal vessels display more tortuosity, especially in layers containing neuronal cell bodies, such as the stratum pyramidale.^16^ String vessels, defined as small 1-µm-diameter collagenous vascular structures, marked by anti-Collagen-IV, are found in regions of abnormal angiogenesis and micro-vascularity of HS+ TLE patients.^95^ Similar to sprouts in the laminal basement membrane in pilocarpine animals, these string vessels do not have a clear lumen nor endothelial lining.^95^ Kastanauskaite et al observed spine-like protrusions, in CA1 of HS+ TLE patients using the same anti-collagen-IV staining method.^94^ Interestingly, reactive astrocytes have been found in the lumen of these protrusions.^94^ The etiology of string vessels is still debated: they are hypothesized to be either collapsed capillaries, and therefore represent vascular degeneration, or may be the result of pericyte basement membrane protrusions originating from abnormal angiogenesis.^50^

## Microcirculation in human epilepsy

In normal physiological circumstances, pial arteries maintain regional cerebral blood flow within a range independent of arterial pressure alterations. This process is called autoregulation. Simultaneously, parenchymal arterioles continuously adapt local cerebral blood flow to changes in neuronal activity and its accessory metabolism.^97^ This means that when neuronal activity and local metabolism increase, local cerebral blood flow increases accordingly to deliver sufficient nutrients. This mechanism is called neurovascular coupling (NVC) and is exerted by the interplay of members of the neurovascular unit (NVU) consisting of neurons, astrocytes, pericytes and endothelial cells.^98,99^

Seizures lead to a simultaneous and severe challenge of both mechanisms of autoregulation and NVC. First of all, blood pressure and heart rate can quickly rise to extreme levels during seizures, thereby challenging autoregulatory responses of cerebral arteries.^100^ Secondly, seizures increase local neuronal activity heavily, leading to a simultaneous and enormous increase of metabolic burden of the involved brain areas. Consequently, local cerebral blood flow should be increased accordingly to provide the required substrates. A recent preclinical study described indeed an increase of both excitatory and inhibitory neuronal activity within the epileptic focus, and a similar increase in vascular diameter and cerebral blood flow.^101^ Interestingly, they also found a pre- and interictal reduction of focal vessel diameter and cerebral blood flow which paralleled a decrease of basal neuronal activity. These findings suggested that the preictal level of vascular and neuronal activity could predict the severity of the upcoming ictal event. Furthermore, pre-ictal vasoconstriction of pial arteries feeding perifocal tissue has been described in chronic focal epilepsy, whereas a late ictal increase of cerebral blood flow has been noted within the epileptic focus.^102^ Furthermore, longitudinal changes in hemodynamic responses to seizures during the course of recurrent seizures have shown that individual vasodilation-constriction responses erode in both capillaries and small cortical arteries. Recently, Zhang et al. suggested that the calcium concentration ([Ca^2+^]) in astrocytic endfeet may regulate parenchymal arterial diameter.^103^ In line with the above-mentioned vasoactive responses, they found an ictal vasodilation within the epileptic focus, whereas a vasoconstriction was noted in the perifocal parenchyma. Although different vasoactive responses were found in the focal and perifocal tissue, an increase of astrocytic endfeet [Ca^2+^] was reported at both sites. This suggests dysfunction of the neurovascular coupling mechanism in both regions. In addition, the authors observed a slow increase of [Ca^2+^] following repetitive seizures in astrocytic endfeet. This resulted in a stronger arteriolar constriction in the focal and perifocal tissues, which is in line with the reduced interictal vascular and neuronal activity as described by Lim et al.^101^ Using 2-photon [Ca^2+^] imaging, it has been shown that seizures result in vasoconstriction of cortical penetrating arterioles. This postictal vasoconstriction in the NVU was associated with seizure-induced hypoxia.^104^ This process has been ascribed to neurovascular decoupling due to failure of one or multiple NVU members to effectuate vasodilation in response to increased neuronal activity.^41^ This might be related to alterations in capillary BBB permeability and perivascular cellular injury of parenchymal arterioles. Excessive vasoconstriction of parenchymal arterioles due to neurovascular decoupling may result in an unintended reduced cerebral blood flow in the downstream capillary bed.^105^ This may lead to a disturbed parenchymal homeostasis in these patients, which on itself forms a substrate facilitating seizures.^105^ Furthermore, as a byproduct of enhanced energy metabolism, oxygen-centered free radicals are formed, leading to oxidative damage of mitochondrial enzymes and DNA. These reactive oxygen-species damage neurons and pericytes, leading to disturbances of NVC, which might explain alterations of the NVU found in epileptogenic areas.^105^ Recent literature has drawn a parallel with traumatic brain injury, in which hyperexcitability and ischemia led to spontaneous seizures, enhanced susceptibility to chemo-convulsants, metabolic stress, inflammatory responses, BBB breakdown, and cell death.^106^ Another aspect to consider here is that a seizure-induced raise of cerebral blood flow alone might not be sufficient to deliver adequate nutrients. This raised cerebral blood flow will mainly increase red blood velocity if the capillary density has not proportionally increased as well. On one hand, the increased red blood velocity itself affects the time available for delivery and exchange of vital nutrients. On the other hand, the lack of additional capillaries has no effect on the total capillary surface available for nutrients exchange and does not alter the distance between brain cells to capillaries. Hence, an additional increase in vessel density, i.e. formation of new capillaries, would accommodate these adaptive processes. Formation of new capillaries requires a key role for VEGF.

Although the potential pathophysiological contribution of the above-mentioned microcirculatory alterations to NVC, the NVU and their relation to TLE awaits further study, the reported pre-ictal, ictal, and interictal changes in vascular and neuronal activity are commonly used to aid the localization of epileptogenic foci in drug-resistant epilepsy patients. Modern imaging techniques like, blood oxygen level-dependent functional magnetic resonance imaging, positron emission tomography, and single-photon emission computerized tomography, are applied in the work-up of drug-resistant epilepsy patient and evaluate various aspects of vascular and neuronal activity.^101,107^

## Summary

In this review, we have summarized the current knowledge on microvascular changes in epilepsy based on animal and human studies. Increased VEGF concentrations have been observed in hippocampi exposed to seizures. Unlike previous reports, a hypoxic environment is not required for VEGF activation; neuronal activity alone seems sufficient. In the epileptic brain, VEGF plays a crucial role in angiogenesis. This VEGF-induced angiogenesis may well be associated with BBB leakage, caused by a mismatch between angiogenesis and barriergenesis.

VEGF-induced angiogenesis also appears to play an important role in MVD increase, as seen in the hippocampus, amygdala, and neocortex in the chronic phase of rodent epilepsy models. In human TLE, an increased hippocampal MVD might be expected, but is not repeatedly found. This might be due to the variety of staining methods used.

Finally, clear microvascular morphological changes are seen in the epileptic hippocampi of patients and animal models including a decrease in afferent vessels and increase in basement membranes. Basement membrane deviations like string vessels and protrusions might be the result of the misbalance between angiogenesis and barriergenesis in epilepsy. Microvascular changes might also affect microcirculatory physiology, with neurovascular decoupling leading to relative hypoperfusion and disturbed parenchymal homeostasis, possibly contributing to epilepsy, although this topic has only scarcely been assessed to date.

## Future perspectives

Although there seems to be a relation between microvascular abnormalities and epilepsy, both in rodents and epilepsy patients, it remains the question of all abnormalities are present in patients. Furthermore, a number of observations mentioned in this review raise questions. What causes the increased vessel diameter in these microvascular alterations? Why is a decrease in afferent vessels found in the hippocampi of TLE patients? Does thrombus formation occur after epileptic seizures, and what is the connection with cerebrovascular disease? To answer these questions a different approach to epileptogenesis and epilepsy research is desirable. Processes of angiogenesis and barriergenesis seem of major interest. In particular, in vivo assessment of cerebral microcirculation could reveal functional alterations evolving from the structural microvascular abnormalities in epilepsy.

## Conclusion

This review of microvascular changes in experimental epilepsy and TLE suggests that epilepsy may well be a disorder associated with disturbed structure and function of the cerebral microvasculature. Several mechanisms of seizure-induced angiogenesis are discussed, as well as angiogenesis-induced vessel leakage, with VEGF as a possible key player. However, investigating cerebrovascular properties and structures during epileptogenesis in humans is extremely difficult, as epilepsy patients already suffer from end-stage disease. The ultimate goal of epilepsy treatment is seizure prevention. Based on this review, assessment of cerebral microvasculature in relation to epileptogenesis and epilepsy might enable new therapeutic targets. In vivo assessment of the cerebral microcirculation could reveal functional alterations evolving from structural microvascular abnormalities in epilepsy. Even though suppression of angiogenesis appears to be anticonvulsive, it is too early to develop treatment aimed at angiogenesis suppression at this moment. However, we may consider epilepsy patients as cerebrovascular patients, and start to emphasize the importance of healthy vascularity.
